# Sleep quality and mood symptoms in conscripted frontline nurse in Wuhan, China during COVID-19 outbreak: A cross-sectional study

**DOI:** 10.1097/MD.0000000000020769

**Published:** 2020-06-26

**Authors:** Zhi-hao Tu, Jing-wen He, Na Zhou

**Affiliations:** a Department of Nautical psychology; b Department of medical psychology; c Mental health education center, the Second Military Medical University, Shanghai, China.

**Keywords:** anxiety, COVID-19, depression, nurse, sleep

## Abstract

The aim of this study was to investigate the prevalence of sleep problems, depression and anxiety symptoms among conscripted frontline nurses fighting coronavirus disease 2019 (COVID-19) in Wuhan.

This study was a cross-sectional study conducted with 100 frontline nurses. Sleep quality, depression, and anxiety symptoms were measured using the Pittsburgh sleep quality index (PSQI), the Generalized Anxiety Disorder 7-Item Scale (GAD-7) and the Patient Health Questionnaire-9 (PHQ-9), respectively.

Mean sleep duration was 5.71 hours (SD = 1.09) and mean sleep latency was 33.49 minutes (SD = 28.87). A total of 76%, 81%, 45%, and 19% reported difficulty initiating sleep (DIS), difficulty maintaining sleep (DMS) or early morning awakening (EMA), nightmares and using hypnotics respectively. Among 100 participants in this study, 60 (60%) had poor sleep quality, 46 (46%) suffered depression symptoms and 40 (40%) reported anxiety symptoms. Sleep quality (OR = 3.16, 95% CI: 1.17–8.52) and anxiety symptoms (OR = 8.07, 95% CI: 2.92–22.33) were significantly associated with depression symptoms. Depression symptoms (OR = 7.92, 95% CI: 2.89–21.73) were related to anxiety symptoms. Similarly, depression symptoms (OR = 3.24, 95% CI: 1.19–8.79) were associated with poor sleep quality.

Sleep disturbance, depression, and anxiety symptoms are very common among frontline nurses who treating patients with COVID-19 in Wuhan, China. Comprehensive measures that involve psychosocial and personal behaviors should be implemented to improve sleep quality and prevent depression and anxiety symptoms.

## 1 Introduction

In December 2019, several cryptogenic cases of pneumonia were reported in Wuhan, Hubei Province, China.^[1]^ A novel coronavirus was isolated from patients with pneumonia,^[2]^ called severe acute respiratory syndrome coronavirus 2 (SARS-CoV-2).^[3]^ On 11 February, 2020, the World Health Organization (WHO) officially named the pneumonia as coronavirus disease 2019 (COVID-19).^[4]^ Epidemiological findings showed that SARS-CoV-2 could be spread by human-to-human transmission via droplets or direct contact.^[3,5]^ It was estimated that the basic reproduction number (R0) of COVID-19 ranged from 2.24 to 3.58.^[6]^ According to authoritative information from the National Health Commission (NHC) of China,^[7]^ 47,441 COVID-19 cases had been confirmed in Wuhan city by February 25, 2020. Such a large number of patients put great pressure on local medical institutions in Wuhan. To better fight COVID-19, the Chinese government mobilized medical workers from all over the country to support Wuhan. There had been more than 42,000 conscripted frontline medical workers from other provinces in Wuhan by February 29, 2020, among whom 68% were nurses.^[8]^ They made great achievements in treatment and control of COVID-19. However, as early as February 11, 2020, 1080 conscripted frontline medical workers in Wuhan had been confirmed with COVID-19, severe cases accounted for 17.7%.^[9]^ Besides high-risk susceptible to COVID-19, conscripted frontline nurses also faced with unfamiliar working and living environment, a shortage of medical supplies, prolonged working hours due to a lack of staff, being separated from family, and lack of social support. These may bring huge physical and mental stress to conscripted frontline nurses and further lead to emotional and sleep problems.

Therefore, in this study, we aimed to investigate the prevalence of sleep problems, depression, and anxiety symptoms among conscripted frontline nurses fighting COVID-19 in Wuhan.

## 2 Materials and methods

### 2.1 Study design and participants

The data were collected between February 7 and February 25, and participants were cluster sampled from “Huoshenshan” Hospital. The inclusion criteria were nurse and treating patients infected with COVID-19. The participants were asked to complete the self-administered questionnaires through mobile phone Wechat. It took approximately 15 minutes to complete the questionnaires. Participants whose response time was less than 3 minutes or more than 30 minutes were excluded to ensure the quality of questionnaires. This study was approved by the Ethical Committee of the Second Military Medical University.

### 2.2 Measures

#### 2.2.1 Sociodemographic variables

The sociodemographic variables include age, sex, working years, education level, marital status, whether the only child in your family and whether have any children.

#### 2.2.2 Sleep quality measure

The Pittsburgh Sleep Quality Index-Chinese version (PSQI)^[10]^ is a 19-item self-report questionnaire which is used to measure sleep disturbances and quality over the past month. The total score comprises 7 component scores, which include subjective sleep quality, sleep duration, sleep latency, sleep disturbances, sleep efficiency, daytime dysfunction, and the use of sleeping medication. Scores range from 0 to 21. A higher score represents worse sleeping conditions, while a low score denotes good sleeping conditions. A score of ≥7 was the cut-off value to indicate poor sleep quality in Chinese population.

#### 2.2.3 Anxiety measure

Generalized Anxiety Disorder 7-Item Scale (GAD-7) was used to assess anxiety symptoms. Patients were asked to rate how often they have been bothered by the described symptoms over the last 2 weeks using a 4-point rating scale from 0 (not at all) to 3 (every day). Total scores range from 0 to 21, with higher scores reflecting higher severity levels of generalized anxiety disorder symptomology. It has good reliability (Cronbachs a = 0.89), as well as criterion, construct, factorial, and procedural validity.^[11]^ A score of ≥4 was the cut-off value to indicate anxiety symptoms in Chinese population.

#### 2.2.4 Depression measure

Depressive symptoms were measured using the Patient Health Questionnaire-9 (PHQ-9).^[12]^ This questionnaire comprises 9 items with scores between 0 and 27. At a score ≥ 10, the PHQ-9 has a sensitivity and a specificity of 88%, and a positive likelihood ratio of 7.1 to detect major depression. The PHQ-9 has demonstrated high internal consistency with Cronbachs α coefficient of 0.87 in the present study. A score of ≥4 was the cut-off value to indicate depression symptoms in Chinese population.

### 2.3 Statistical analysis

Statistical analysis was performed with SPSS version 22.0. Descriptive statistics such as mean, range, standard deviation (SD), and percentages were calculated. Expectation maximization algorithm was used to fill in missing data if existed. Multivariate logistic regression analyses were used to calculate the crude and adjusted odds ratio (OR) with 95% confidence interval (CI) to determine the association between different socio-demographic variables, anxiety, depression, and sleep quality. For all tests, values of *P* ≤ .05 were considered statistically significant.

## 3 Result

### 3.1 Sample characteristics

Response rate was 100%. The mean age of 100 frontline nurse was 34.44 (SD = 5.85, ranging from 21 to 46 years). All participants were female. Most participants have a college degree (83%). Seventy frontline nurses were married (70%) and most of them have children (66%) (Table 1).

**Table 1:**
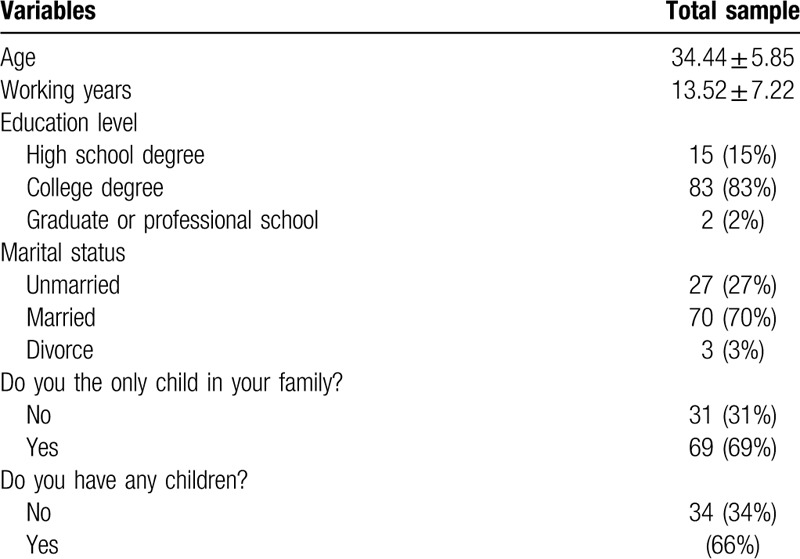
Descriptive data of 100 frontline nurses.

### 3.2 Sleep disturbance of 100 frontline nurses

We found sleep duration was 5.71 hours (SD = 1.09). A total of 34%, 43%, 19%, and 4% reported sleeping ≥5, 5-6, 6-7, and >7 hours, respectively. Sleep latency was 33.49 minutes (SD = 28.87). In addition, Seventy six percent of nurses reported symptoms of difficulty initiating sleep (DIS). Most nurses have difficulty maintaining sleep (DMS) or early morning awakening (EMA). About half of participants have nightmares (45%). Nineteen percent of nurses used hypnotics during their treating patients with Coronavirus Disease 2019 (COVID-19) in Wuhan, China. (Table 2)

**Table 2:**
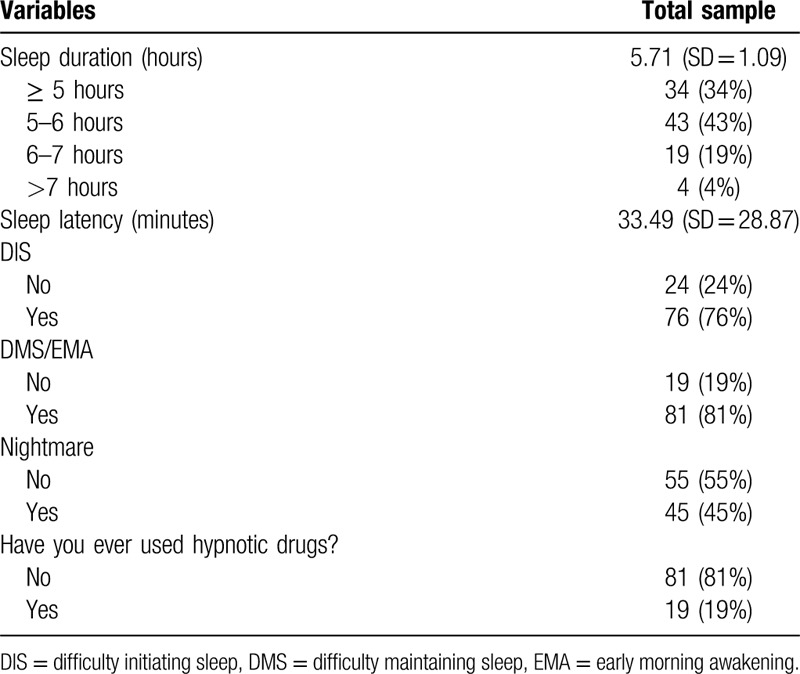
Sleep disturbance of 100 frontline nurses.

### 3.3 Prevalence of depression, anxiety, and sleep quality in 100 frontline nurses

PSQI score was 8.48 (SD = 3.63, range from 2 to 18). The prevalence of poor sleep quality was 60%. PHQ-9 score was 4.64 (SD = 3.48, range from 0 to 20) and 46 nurses (46%) might have depression symptoms. Similarly, GAD-7 score was 4.05 (SD = 3.83, range from 0 to 20) and 40 participants (40%) might have anxiety symptoms (Table 3).

**Table 3:**
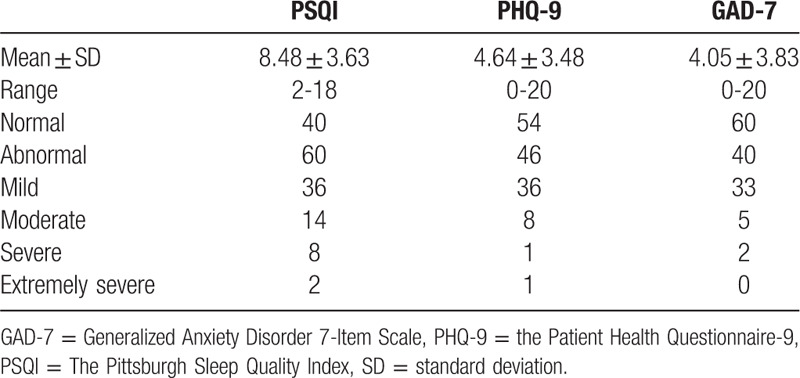
The prevalence, degrees and scores of depression, anxiety, and sleep quality in 100 frontline nurses.

### 3.4 Association between socio-demographic variables, anxiety, depression, and sleep quality

The associations of potential influence factors with PHQ-9, GAD-7 and PSQI were presented in Table 4. In the multivariate logistic regression models, sleep quality (OR = 3.16, 95% CI: 1.17–8.52) and anxiety symptoms (OR = 8.07, 95% CI: 2.92–22.33) were significantly associated with depression symptoms. In addition, a marginal significance was found between participant who was only child in her family (OR = 0.36, 95% CI: 0.12–1.04) and depression symptoms. Depression symptoms (OR = 7.92, 95% CI: 2.89–21.73) were related to anxiety symptoms. Similarly, depression symptoms (OR = 3.24, 95% CI: 1.19–8.79) were associated with poor sleep quality.

**Table 4:**
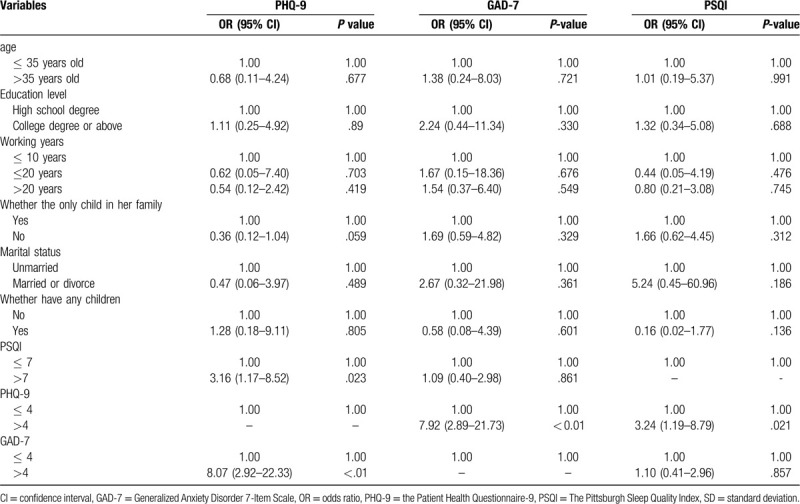
Multivariate logistic regression analyses between socio-demographic variables, anxiety, depression, and sleep quality.

## 4 Discussion

The results showed that frontline nurses had short sleep duration, long sleep latency, and insomnia symptoms. Among the 100 participants, 60 nurses (60%) had poor sleep quality, 46 nurses (46%) developed symptomatic depression, and 40 participants (40%) might have anxiety symptoms. In addition, sleep quality and anxiety symptoms were significantly associated with depression symptoms. Depression symptoms were related to anxiety symptoms. Depression symptoms were associated with poor sleep quality.

The average PSQI score of frontline nurses in this study was 8.48 ± 3.63, which was much higher than 7.32 ± 3.24 of nurses in general hospitals in China measured at ordinary times.^[13]^ A meta-analysis of prevalence of sleep disturbances in Chinese healthcare professionals showed that the pooled prevalence is 39.2% (95% CI: 36.0%–42.7%), which is far lower than 60.0% in our study.^[14]^ Similarly, the rate of difficulty initiating sleep (DIS), difficulty maintaining sleep (DMS), early morning awakening (EMA) were higher in conscripted frontline nurses than average Chinese nurses.^[15]^ The following reasons might account for higher prevalence of sleep disturbance in frontline nurses in Wuhan. First, the study was conducted at the initial stage of COVID-19 outbreak, when there was a shortage of medical workers (especially nurses) in Wuhan. Thus, conscripted frontline nurses faced with huge stress at work and prolonged working hours. The heavy workload might lead to poor sleep quality.^[16]^ Second, perceived negative feelings like fear of being infected and passing COVID-19 to their friends and colleagues and lack of knowledge about COVID-19 might result in sleep disturbance.^[17]^ Third, some participants in this study reported that missing their family was one of the main factors influencing their sleep quality. In fact, most conscripted frontline nurses in “Huoshenshan” Hospital were far away their family and hometown in other provinces. These conditions might lead to deprivation of social support, which may further cause sleep disturbance.^[18]^

The mood status of conscripted frontline nurses was also not optimistic. Interestingly, although the prevalence of depression and anxiety symptoms reached 40% and 46%, the prevalence of depression and anxiety symptoms in medical workers during Severe Acute Respiratory Syndromes (SARS) outbreak was much higher,^[19]^ even the prevalence in Chinese nurses during ordinary times was higher.^[20,21]^ Actually, most conscripted frontline nurses in Wuhan were volunteered to support Wuhan, while some nurses in previous studies during SARS outbreak were conscripted involuntarily.^[22]^ Thus, conscripted frontline nurses were more prepared to the difficulties in Wuhan both mentally and physically. On the other hand, supporting Wuhan to fight COVID-19 was a glorious mission, and conscripted frontline nurses were regarded as heroes in China. These conditions may be related to higher level of job satisfaction and more positive attitude, which may explain why the prevalence of mood symptoms among them was lower.^[17,21]^ But it should not be ignored that there still were a fairly high percentage of conscripted frontline nurses had mood symptoms, especially when taking the considerable total number of nurses in Wuhan into account. The anxiety and depression symptoms may be due to lack of social support, stress at work, worry about COVID-19, and perceived negative feelings mentioned above.^[17,23–26]^

In this study, we did not find any significant association between sociodemographic variables (age, education level, working years, marital status, etc.) and sleep disturbance or mood symptoms. However, previous studies suggested that there was a trend of increasing report of mood symptoms and sleep disturbance with lower age and shorter working years in nurses.^[15,20,21]^ A study on frontline nurses during SARS outbreak also showed that lower age (<29 years) was associated with affective disorder and insomnia.^[17]^ Lower age and shorter working years mean lack of experience and knowledge on treating patients with pandemic like A/H1N1 influenza or COVID-19, which may be related to higher degree of worry about the pandemic and further lead to psychological distress.^[26]^ However, before leaving for Wuhan, every nurse learned most recently updated knowledge about COVID-19 and detailed procedure of treating the disease. This may reduce the negative effect of lack of knowledge and experience and explain the insignificant association between lower age and shorter working years. This study also found that sleep disturbance, anxiety symptoms, and depression symptoms were related to each other in conscripted frontline nurses. This result was consistent with previous studies.^[27,28]^

Several limitations of the present study must be addressed. First, this was a cross-sectional study. Thus, the relationships between socio-demographic variables, anxiety, depression, and sleep quality cannot be determined in present study. Second, the present study used PSQI to measure the nurses sleep, which might not be very objective. Nurses are shifts workers, so their sleep might be worse than reported. Third, the sample size was small, thus limiting the interpretation of the results. These findings might set the basis for future studies.

## 5 Conclusion

Sleep disturbance, depression, and anxiety symptoms are prevalent among frontline nurses who treating patients with COVID-19 in Wuhan, China. The results of this research suggest comprehensive measures that involve psychosocial and personal behaviors should be implemented to improve sleep quality and prevent depression and anxiety symptoms.

## Acknowledgments

The authors thank the participants and other nurses in Huoshenshan Hospital. You are heroes and heroines!

## Author contributions

**Conceptualization:** Jing-wen He, Na Zhou.

**Data curation:** Na Zhou.

**Formal analysis:** Jing-wen He.

**Investigation:** Jing-wen He.

**Methodology:** Jing-wen He.

**Supervision:** Na Zhou.

**Software:** Jing-wen He.

**Writing – original draft:** Jing-wen He.

**Writing – review & editing:** Jing-wen He, Na Zhou.
