# Erratum

**DOI:** 10.1097/MD.0000000000022948

**Published:** 2020-10-16

In the article, “Sleep quality and mood symptoms in conscripted frontline nurse in Wuhan, China during COVID-19 outbreak: A cross-sectional study”,^[1]^ which appears in Volume 99, Issue 26 of *Medicine*, the data in Table 3, column PHQ-9, rows mild, moderate, severe and extremely severe were incorrect. The correct table is below.

**Table 3:**
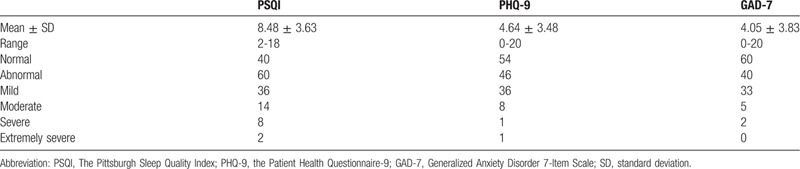
The prevalence, degrees and scores of depression, anxiety and sleep quality in 100 frontline nurses.
